# Potentially Inappropriate Medication and Polypharmacy in Nursing Home Residents: A Cross-Sectional Study

**DOI:** 10.3390/jcm11133808

**Published:** 2022-06-30

**Authors:** Raquel Díez, Raquel Cadenas, Julen Susperregui, Ana M. Sahagún, Nélida Fernández, Juan J. García, Matilde Sierra, Cristina López

**Affiliations:** 1 Pharmacology, Department of Biomedical Sciences, Faculty of Health Sciences, Institute of Biomedicine (IBIOMED), University of León, 24071 León, Spain; rdielz@unileon.es (R.D.); rcads@unileon.es (R.C.); nferm@unileon.es (N.F.); jjgarv@unileon.es (J.J.G.); msiev@unileon.es (M.S.); clopcd@unileon.es (C.L.); 2 Applied Mathematics, Department of Mathematics, University of León, 24071 León, Spain; jsusl@unileon.es

**Keywords:** 2019 Beers criteria, drug–drug interactions, elderly, nursing home, polypharmacy, potentially inappropriate medication, PRISCUS list, v2 STOPP/START criteria

## Abstract

Inappropriate prescribing in the elderly is a risk factor for higher adverse drugs reactions, hospitalisation, and mortality rates. Therefore, it is necessary to identify irrational prescriptions and implement interventions to improve geriatric clinical practices in nursing homes. This study aimed to examine and compare the prevalence of potentially inappropriate medications in nursing home residents using three different updated criteria: 2019 Beers criteria, PRISCUS list, and v2 STOPP criteria, and to determine the prevalence of potential prescribing omissions according to v2 START criteria. A descriptive, observational, and cross-sectional study design was used. A total of 218 residents were involved in this study. Data on drug use were collected from medical charts. Information was screened with the software CheckTheMeds. Potentially inappropriate medications were present in 96.3%, 90.8%, and 35.3% of residents, according to the STOPP, Beers, and PRISCUS criteria or list, respectively. Inappropriate medication was found to be significantly associated with polypharmacy and severe or moderate drug–drug interactions with the three tools and with pathologies and unnecessary drugs only for STOPP criteria. The most frequent inappropriate medications were benzodiazepines and proton pump inhibitors. A regular use of software to review medications in nursing home residents would help to reduce the risk of these drug-related problems.

## 1. Introduction

People around the world are living longer than ever. By 2030, one in six people in the world (1.4 billion) will be aged 60 and over, whereas the number of people aged 80 and over is expected to triple up to 426 million between 2020 and 2050 [1]. In Spain, the population of people over 65 years amounts to 9.38 million individuals, almost one-fifth of the Spanish population [2]. According to Eurostat projections, by 2050, four Spanish provinces will be among the 10 most aged regions in the European Union (EU) [3]. In line with this increase, efforts have also been intensified to evaluate the health problems of the elderly to mitigate and prevent them.

The prescription of potentially inappropriate medication (PIM) is one of these health problems faced by the geriatric population [4,5,6,7]. PIMs may be defined as drugs for which use among older adults should be avoided due to the high risk of adverse reactions for this population and/or insufficient evidence of their benefits when safer and equally or more effective therapeutic alternatives are available [8]. Monitoring PIM use in an ageing population is critical, as it is related to multimorbidity and polypharmacy. Consequently, the risk of adverse drug reactions, drug–drug interactions (DDIs), and drug–disease interactions with complex medication regimens increases. These drug-related problems can worsen patient health, as PIMs are also associated with higher hospitalisation and mortality rates in older patients [9,10]. In Spain, the number of beds available in nursing homes (NHs) is 4.1% of people aged 65 or more [11]. In these settings, it is characteristic to observe a high turnover of nurses, especially after pandemic, and the absence of a pharmacist in most of them, which may make medication follow-up difficult.

Different strategies have been designed to deal with inappropriate prescription. Numerous criteria are currently available to reduce the number of PIMs in the elderly. They can be categorised into implicit (judgement-based) and explicit (criterion-based) tools. Some explicit tools are specifically used for PIM screening, such as the Beers criteria [12], the European Union (7)-PIM list [13], or the PRISCUS list [14]. Other criteria evaluate PIMs and potential prescribing omissions (PPOs) such as the Screening Tool of Older People’s Prescriptions (STOPP) or the Screening Tool to Alert to Right Treatment (START) criteria [15]. STOPP and START criteria were the first European explicit criteria and are the most used and validated among the European elderly population. After version 1 (84 criteria) [16], version 2 was developed (114 criteria), expanding the explicit criteria as well as incorporating three implicit criteria [15]. The Beers criteria are the most important tool in the USA, managed since 2011 by the American Geriatrics Society (AGS) and updated on a 3-year cycle that began in 2012 [12,17,18]. Finally, the PRISCUS list was created for the German pharmaceutical market but has not been updated since 2010 [14].

Although assessing the appropriateness of prescribing medication in NH patients should be mandatory, there is a lack of data on the prescribing patterns of PIMs and PPOs in this ageing population in Spain. Therefore, the aim of this study was to examine and compare the prevalence of PIMs in NH residents using three different updated criteria for PIM: 2019 Beers criteria, PRISCUS list, and v2 STOPP criteria. We also tried to determine the prevalence of PPOs according to v2 START criteria. Finally, the relationships between PIMs and PPOs with polypharmacy and other factors were also evaluated.

## 2. Materials and Methods

A descriptive, observational, and cross-sectional study was performed in an NH of the region of Leon, one of the most aged in Spain. It was conducted from August to December 2021. The target population was NH residents aged 70 years or older. Information on the institutionalised elderly and their treatments were carefully recorded from the NH management software and completed with supplementary clinical information obtained from the NH physician. Collection was carried out guaranteeing the anonymity of the patients and the confidentiality of the data. The Strengthening the Reporting of Observational Studies in Epidemiology (STROBE) Statement was used to report data [19].

Demographic characteristics (age and sex), pathologies (International Classification of Diseases, Tenth Revision, ICD-10), and the Charlson comorbidity index (CCI) [20] of the NH residents were registered. Medications included chronic treatments administered by oral, inhalation, or ophthalmic routes. All of them required a prescription and had been administered to residents for at least 1 month prior to data collection. Over-the-counter (OTC) medicines, dietary supplements, or herbal medicines were excluded as it was not possible to document them for each patient. All treatments were classified according to the World Health Organization (WHO) anatomical therapeutic chemical (ATC) classification system [21]. Any combination medicine (multicomponent products) was considered a single medicine.

According to previous studies, polypharmacy status was categorised into 3 groups: non-polypharmacy (0–4 medicines), polypharmacy (5–9 medicines), and excessive polypharmacy (≥10 medicines) [22,23,24,25,26]. The 2019 Beers criteria, the PRISCUS list, and v2 STOPP criteria were used to classify medications as PIM, and the v2 START criteria were used for PPO.

The information obtained was evaluated with CheckTheMeds (CheckTheMeds v.3.6.4, CheckTheMeds Technology SL, Almería, Spain). This software is routinely used in hospitals to process individual patient information by combining clinical and pharmacological data to detect drug-related problems, such as duplicity and DDIs, and has several automated criteria or lists such as STOPP, START, Beers, and PRICUS to detect PIMs. An academic pharmacist and a geriatric nurse counterchecked the results provided by each NH resident.

The necessary minimum sample size was estimated as 171 residents assuming a precision of 0.075, an estimated probability of 0.5, and a significance level of 0.5 [27]. The NH was chosen as it exceeded the minimum sample size, to obtain better precision.

### 2.1. Statistical Analysis

Data analysis was performed with the statistical package IBM SPSS Statistics 26 (IBM Corporation, Armonk, NY, USA). Descriptive statistics (frequencies, median, standard deviations, ranges, and percentages with 95% confidence intervals) were used to characterise the study population.

Logistic regression was performed to identify those demographic and clinical variables potentially associated with PIMs according STOPP, START, Beers, and PRISCUS criteria or list. Odds ratios (ORs) were calculated with their respective 95% confidence intervals (95% CI). Multivariable forward-step ordinal logistic regression analysis was conducted to assess the impact of each predictor on PIM.

Agreement between criteria used to identify PIMs was estimated with kappa statistics (values of kappa >0.75 indicate good to excellent agreement; 0.40–0.75 moderate agreement; <0.40 poor agreement [28]). Spearman correlation (ρ) was also employed to analyse the association between criteria.

A *p*-value of <0.05 was always considered as significant.

### 2.2. Ethical Considerations

The study was approved in advance by the Institutional Review Board of the Nursing Home and the Ethics Committee of the University of Leon (ULE-015-2021) and carried out in accordance with the Declaration of Helsinki.

## 3. Results

A total of 218 NH residents were included in the present study with a mean age of 85.9 ± 7.4 years (range 70–107; median 86). Table 1 summarises the demographic and clinical characteristics of the participants. All NH residents showed multimorbidity (two or more chronic conditions). The most common chronic diseases were hypertension (55.8%), cognitive impairment (35.1%), and cataracts (29.1%).

Polypharmacy was present in 79.8% of NH residents, who consumed a total of 1535 drugs with a median of 7 (range = 1 to 17). Regarding unnecessary drugs, we detected 466 in 191 participants (87.6%; median = 2; range = 0 to 10). We also identified a total of 359 severe or moderate DDIs in 119 NH residents (54.6%) with a median of 1 DDI per participant, ranging from 0 to 17.

Table 2 shows the demographic and clinical characteristics of the NH residents related to the occurrence of any PIMs and PPOs according to STOPP, START, Beers, and PRICUS criteria or list.

### 3.1. PIM According to STOPP Criteria

A total of 38 different types of PIMs were identified with STOPP criteria. No PIM was detected in only eight NH residents (3.7%; 95% CI: 1.2–6.2). Of the remaining 210 participants, 31 (14.2%; 95% CI: 9.6–18.9) had only one PIM; 38 (17.4%; 95% CI: 12.4–22.5) received two PIMs; 30 (13.8%; 95% CI: 9.2–18.3) had three PIMs; 31 (14.2%; 95% CI: 9.6–18.9) had four PIMs, and more than a third (36.7%; 95% CI: 30.3–43.1) had five PIMs.

Among those 1535 drugs prescribed, 852 (55.5%; median 2; range 0–15) were classified as PIM based on STOPP criteria. The section with the highest number of PIMs was A (indication of medication) with 475, followed by D (central nervous system and psychotropic drugs) with 143 and K (drugs that predictably increase the risk of falls in older people) with 103.

The top five PIMs identified belonged to criteria A1 (any drug prescribed without an evidence-based clinical indication) with a total of 55.8% (range 0–10); criteria D5 (benzodiazepines for ≥4 weeks) with a total of 12.9% (range 0–3); criteria K1 (benzodiazepines) with 7.9% (range 0–2); criteria K2 (neuroleptic drugs) with 3.2% (range 0–2); and F2 (PPIs for uncomplicated peptic ulcer disease or erosive peptic oesophagitis at full therapeutic dosage for >8 weeks) with 2.7% (range 0–3). The presence of any PIM related to benzodiazepines in STOPP criteria A1 (any drug prescribed without an evidence-based clinical indication), D5 (benzodiazepines for ≥4 weeks), G5 (benzodiazepines with acute or chronic respiratory failure), and K1 (benzodiazepines) was found in 30.4%.

The results of the multivariate ordinal logistic regression analysis relevant to a higher PIM according to STOPP criteria are presented in Table 3.

### 3.2. PIM According to Beers Criteria

Less than 1 in 10 participants had zero PIM (9.2%; 95% CI: 5.3–13.0). Of the remaining 198 NH residents (90.8%), 30 (13.8%; 95% CI: 9.2–18.3) were prescribed only one PIM, 39 (17.9%; 95% CI: 12.8–23.0) used two PIMs, 24 (11.0%; 95% CI: 6.9–15.2) had three PIMs; 19 (8.7%; 95% CI: 5.0–12.5) had four PIMs, and more than a third (39.4%; 95% CI: 33.0–45.9) received five PIMs.

Of the 1535 drugs prescribed, 927 (61.0%; median 3; range 0–18) were classified as PIM based on Beers criteria according to 42 different items. More than two-thirds of the PIMs detected (69.5%) should be avoided in older adults according to Beers criteria, and almost all of the other third (30.3%) should be used with caution.

Regarding DDIs, 23.3% of the detected PIMs were classified as potentially clinically important DDIs to be avoided in older adults. Of these, the most frequent one was the combination of three or more CNS-active drugs (87.5%).

Of relevance is the high number of PIMs related to proton pump inhibitors (PPIs) (12.2%), whose scheduled use should be avoided for more than 8 weeks, except in high-risk patients, erosive esophagitis, or demonstrated need for maintenance treatment. The number of PIMs related to the possible exacerbation or cause of the syndrome of inappropriate antidiuretic hormone secretion (SIADH) or hyponatremia was also important (28.9%). In this case, the use with caution is recommended.

The results of the multivariate ordinal logistic regression analysis relevant to a higher PIM according to Beers criteria are presented in Table 4.

### 3.3. PIM According to PRISCUS List

More than a half of the NH residents (64.7%; 95% CI: 58.3–71.0) had no PIM according to the PRISCUS list. Of those having one or more PIMs, 52 (23.9%; 95% CI: 18.2–29.5) had only one; 23 (10.6%; 95% CI: 6.5–14.6) received two PIMs, and three or four PIMs were detected in only one person each (0.5%; 95% CI: 0–1.4). For the PRISCUS list, we identified 13 different PIMs.

Of the 1535 drugs prescribed, only 105 (6.8%; median 0; range 0–4) were classified as PIM based on the PRISCUS list. Of these medications, 63.8% were sedative and hypnotic agents, followed by antiarrhythmic (18.1%) and anticholinergic (5.7%) drugs.

Table 5 summarises the results of the multivariate ordinal logistic regression analysis relevant to a higher PIM according to the PRISCUS list.

### 3.4. PPO According to START Criteria

Only 84 NH residents (38.5%) showed no omission in prescription (38.5%; 95% CI: 32.1–45.0); 41 people (18.8%; 95% CI: 13.6–24.0) had one PPO; 53 (24.3%; 95% CI: 18.6–30.0) had two PPOs; 21 (9.6%; 95% CI: 5.7–13.5) had three PPOs; in 13 of them (6.0%; 95% CI: 2.8–9.1), four PPOs were reported; and in 6 (2.8%; 95% CI: 0.6–4.9), five PPOs were identified.

According to START criteria, we detected 25 different PPOs. The section with the highest number of omissions was Section E (musculoskeletal system) with 38.3%, followed by Section A (cardiovascular system) with 33%. The most frequent ones among these patients were criteria E3 (vitamin D and calcium supplement in patients with known osteoporosis, previous fragility fracture(s), and/or bone mineral density (T-scores more than −2.5 in multiple sites)) with 34.3%; criteria A6 (angiotensin-converting enzyme (ACE) inhibitor with systolic heart failure and/or documented coronary artery disease) with 9.7%; and A8 (appropriate beta-blocker (bisoprolol, nebivolol, metoprolol, or carvedilol) with stable systolic heart failure) with 7%.

For these criteria, multivariate ordinal logistic regression analysis revealed that comorbidities were significantly associated with a higher risk of PPO (OR: 2.49; 95% CI: 1.627–3.810; *p* < 0.001).

### 3.5. Comparisons and Correlations between PIM Criteria

Table 6 shows the prevalence rates for PIM and the sensitivity and specificity of the 2019 Beers criteria and PRISCUS list in comparison to the v2 STOPP criteria. These latter criteria were used as the reference standard because they are the most currently used and validated in European elderly population [29]. In this sense, the European Union Geriatric Medicine Society (EUGMS) has supported these criteria since 2011 [30].

Significant Spearman correlations were found between PIMs identified by v2 STOPP and 2019 Beers criteria (ρ = 0.55; *p* < 0.001); v2 STOPP criteria and the PRISCUS list (ρ = 0.41; *p* < 0.001); and 2019 Beers criteria and the PRISCUS list (ρ = 0.36; *p* < 0.001) among the NH residents.

## 4. Discussion

To the best of our knowledge, this is the first study to compare PIM prevalence with the updated version of the most important explicit criteria, STOPP and Beers, with the PRISCUS list. Our results showed a significant relationship between PIMs, polypharmacy, and the occurrence of potential DDIs. The sample of this study was an NH in the region of León, which will become the fourth most aged region in the entire EU according to Eurostat forecasts [3].

Our study revealed a high percentage of NH residents with an elevated multimorbidity (≥6 pathologies) (78.0%) and a high prevalence of polypharmacy (79.8%), as well as unnecessary drugs (87.6%) and potentially severe or moderate DDIs (54.6%). We also detected that most of those residents had at least one PIM according to version 2 STOPP (96.3%) and 2019 Beers criteria (90.8%), whereas detection by the PRICUS list was clearly lower (35.3%). According to START criteria, 61.5% of NH residents had at least one PPO.

The use of PIMs in the elderly is a common problem worldwide. Several studies have reported its high prevalence in prescribed treatments. Our study revealed higher PIM prevalence than those detected in Spain’s hospitalised patients (76.7% with version 2 STOPP and 89.0% with 2019 Beers criteria). Nevertheless, detection with the PRISCUS list was higher in the hospital (41.9%) [31]. Other authors have reported a prevalence of 9.7–73.2% for version 2 STOPP criteria [29,32,33,34,35,36,37] and 68.8–79.3% for 2019 Beers criteria [33,38].

NH residents with five or more drugs (polypharmacy) were prone to a significant number of inappropriate prescriptions using any of these three criteria, and something similar happened with the existence of potential severe or moderate DDIs. These findings are consistent with data previously published for polypharmacy [36,39,40,41] and DDIs [6,42,43].

Other predictors of inappropriate prescribing reported in the literature are age and sex [41], although others have reported mixed results [34,44,45,46]. In our study, age was associated with the occurrence of PIM according to the STOPP criteria, and sex according to the Beers criteria. Pathologies are also relevant, as older adults living in NHs tend to have more comorbidities than their non-institutionalised counterparts [47].

Unnecessary drugs were also a significant predictor to take into account in the detection of PIMs according to the STOPP criteria. In this sense, it should be noted that the highest number of PIMs detected (55.8%) was in criteria A1 (any drug prescribed without an evidence-based clinical indication), which is more than double the value reported by Baré et al. (25.7%) [29]. A1 is a very diverse criterion, and it does not specify which drug is involved in the pharmacological groups. Thus, it should be modified to become more explicit and avoid subjectivity in the screening. After classifying data in criterion A1 into pharmacological drugs, the two most relevant ones were benzodiazepines (16%) and PPIs (13.9%).

An important finding of this work is that about a quarter (21.5%) of the NH residents had a PIM related to benzodiazepines according to the STOPP criteria D5, G5, and K1. Moreover, if all benzodiazepine-related criteria were added together, including those PIMs revealed with A1, it reached 30.4%. Similar values have been reported in previous studies [48,49]. Benzodiazepines are commonly prescribed in the elderly for anxiety and insomnia in spite of being one of the pharmacological groups usually implicated in the occurrence of potential DDIs and adverse reactions (sedation, falls and fractures, mental confusion, cognitive decline, etc.) and in the prescription of unnecessary drugs [26,50,51,52]. If the Beers criteria were applied, benzodiazepines were also involved in 21.6% of PIMs, a value very close to that obtained with the STOPP criteria without the A1 criterion.

As for PPIs, numerous studies have indicated that these drugs are the most frequently overprescribed all over the world [53,54] and that 30–50% of these prescriptions would be inappropriate [55]. In Spain, omeprazole is the most consumed active ingredient in the National Health System [56]. In our study, the F2 STOPP criterion (PPIs for uncomplicated peptic ulcer disease or erosive peptic oesophagitis at full therapeutic dosage for >8 weeks) was implicated in 2.7% of PIMs, and according to the Beers criteria, accounted for 12.2% of PIMs. In the case of the STOPP criteria, data were under-detected if only the F2 criterion was considered. Again, if all PPI-related PIMs identified with the A1 criterion were considered, the prevalence rose to 10.3%. PPIs are drugs also involved in the occurrence of potential DDIs and adverse reactions with relevant clinical consequences for the elderly such as hypomagnesaemia, dementia, or fractures [57,58].

Regarding the Beers criteria, it should be highlighted that of the 69.5% of PIMs that the elderly are recommended to avoid, almost half (49.4%) had high-quality evidence and all of them had a strong strength of recommendation. We also found that 37.2% were related to interactions detected in different sections of the Beers criteria: potentially clinically important DDIs to be avoided in older adults (23.3%) and PIM use in older adults due to drug–disease or drug–syndrome interactions that may exacerbate the disease or syndrome (13.9%). In the case of potentially clinically important DDIs, 87.5% were due to the use of three or more CNS-active drugs, and in most of them (83.1%), combinations including benzodiazepines and nonbenzodiazepines, benzodiazepine receptor agonist hypnotics, or opioids were included, with a remarkably increased risk of falls and other disorders such as central nervous system depression and dementia [26,50,59,60]. A high consumption of benzodiazepines has been reported among NH residents, who also showed an increased risk of mental health disorders [61]. Moreover, this use perhaps has to do with the fact that in 2020, Spain had the highest rate of benzodiazepine consumption worldwide, with 110 S-DDD per 1000 inhabitants per day, a value which should be largely reduced [62]. South Korea has applied a real-time drug utilisation review program for long-acting benzodiazepines and tricyclic antidepressants to patients aged 65 years and above so that a pop-up window opens at the time of the prescription to inform the prescriber [5]. Although diabetes is one of the most common pathologies in the elderly worldwide, PIMs with antidiabetics detected with the STOPP (0.6%) and Beers (0.2%) criteria were very rare in our study. Some authors have reported the potential interaction between metformin and diazepam or PPIs [63,64]. We detected only five PIMs (0.6%) according to the STOPP criteria with metformin (PIM in older people with acute or chronic kidney disease with renal function below particular levels of estimated glomerular filtration rate (eGFR); metformin if eGFR < 30 mL/min/1.73 m^2^ (risk of lactic acidosis)).

The STOPP and Beers criteria identified significantly more PIMs compared with the PRISCUS list. Taking v2 STOPP criteria as the reference standard, the 2019 Beers criteria had higher sensitivity and coefficient of agreement in comparison with PRISCUS but low specificity. The low concordance among criteria has been reported elsewhere [65,66,67]. The use of one or another is related to geographical location (STOPP criteria are more common in Europe, and Beers in the USA), but also with the setting in which they are going to be applied in clinical practice. As for PRISCUS, the lack of updates may have accounted for its low level of detection.

In the present study, 61.5% of NH residents had one or more medications omitted from their treatments according to the START criteria, which is much higher than those reported by other authors at 19.8–57.7% [29,36,37]. Some reasons that may explain this high rate of drug omissions are the need to avoid polypharmacy in an overtreated population, the clinical experience of the physician in charge, and also the existence of comorbidities, which may discourage one from adding more medicine. In fact, comorbidities were a significant predictor of drug omissions according to the START criteria, as in other studies [36]. The most commonly detected PPOs were associated with musculoskeletal system medication, specifically the absence of vitamin D and calcium supplements in patients with known osteoporosis and/or previous fragility fracture, which is in line with Da Costa et al. [68] and Akkawi et al. [69]. Nevertheless, it should be noted that the strong levels of sun radiation in Spain may prevent from their prescription. This is an example of how a list of criteria cannot substitute the clinical judgement of professionals and the individualised approach to patients and treatments. Another important group of prescribing omissions was the cardiovascular system, specially the ACE inhibitor (9.7%) and appropriate beta-blockers (7.0%), but with a lower prevalence than that reported by other authors [69].

Inappropriate polypharmacy is a global problem in the elderly, as it decreases their quality of life and increases medication costs and healthcare system use. In the present study, the prevalence of PIM was 96.3% (v2 STOPP criteria) and 90.8% (2019 Beers criteria), which is much higher than the range reported elsewhere (9.7–79.3%) [29,32,33,34,35,36,37,38]. PIMs should be carefully reviewed for discontinuation, especially when there is evidence of a more effective or safer alternative drug, as it is related to a higher risk of triggering adverse events [13]. In recent years, improving medication prescription in the elderly has received increasing attention. The Spanish Society of Primary Care Pharmacists (SEFAP) has recommended to review medications every 6 months for NH residents with polypharmacy and at least once a year for other institutionalised persons [70]. In addition, it would be necessary to integrate the pharmacist in NHs for interdisciplinary collaboration with physicians and nurses to identify, solve, and prevent drug-related problems [71].

Our study has limitations. The relatively small sample size and the fact that we analysed only one NH means that we cannot generalise our results, but as we already mentioned, the province of León is one of the oldest in Spain and Europe. Moreover, we did not take into account OTC medicines, dietary supplements, or herbal medicines that may be consumed by NH residents and may increase the number of PIMs and DDIs. Moreover, it should also be noted that there are differences in prescribing between regions and countries, especially in the case of the Beers criteria. This was a retrospective study, so the data collected were limited to the information provided in the clinical history, and on occasions, it was not possible to obtain more information on the patient’s condition or pharmacological history prior to admission into the NH.

As strengths, we may include that the PIMs detected were more accurate, as they were defined from the patients’ medical charts and completed with the help of the NH physician. On the other hand, we used three different tools to compare and identify PIMs in the elderly, which clearly improved this analysis. In addition, feedback was provided to NH prescribers to implement appropriate interventions and decrease drug-related problems among residents.

Identification of PIM in NH residents may help to define better prevention strategies and improve the quality of life of this population. Although our findings require further research, they may serve to develop targeting strategies. Establishing a detailed understanding of the patterns and characteristics of potentially inappropriate medication in the elderly may provide a basis for minimising its risk.

## 5. Conclusions

Optimisation of pharmacotherapy has become a global public health problem. This study highlights the need for continuous assessment for prescribed medications to prevent and reduce medication errors, and consequently, their potential adverse drug reactions and DDIs. A very high incidence of PIMs was reported in an institutionalised population, and polypharmacy and the occurrence of potential DDIs were significant predictors of medication inappropriateness. Benzodiazepines and PPIs were the pharmacological groups most frequently involved in PIMs according to v2 STOPP and 2019 Beers criteria.

Polypharmacy and the occurrence of severe or moderate DDIs were significantly associated to the number of PIMs detected in the three tools used, whereas the number of chronic illnesses correlated with prescribing omissions in this group of the population.

Finally, it was evidenced that the routine use of software to check medication would help to reduce the number of PIMs.

## Figures and Tables

**Table 1:** Demographic and clinical characteristics of NH residents (*n* = 218).

Demographic and Clinical Characteristics	Number (%)	95% CI
**Sex**		
Female	145 (66.5)	60.2–72.8
Male	73 (33.5)	27.2–39.8
**Age (years)**		
70–79	48 (22.0)	16.5–27.5
80–89	101 (46.3)	39.7–52.9
≥90	69 (31.7)	25.5–37.8
**Pathologies**		
2–5	55 (25.2)	19.5–31.0
6–10	110 (50.5)	43.8–57.1
≥11	53 (24.3)	18.6–30.0
**CCI**		
3–4	48 (22.0)	16.5–27.5
≥5	170 (78.0)	72.5–83.5
**Polypharmacy**		
Non-polypharmacy	44 (20.2)	14.9–25.5
Polypharmacy	130 (59.6)	53.1–66.1
Excessive polypharmacy	44 (20.2)	14.9–25.5
**Unnecessary drug**		
0 drugs	27 (12.4)	8.0–16.8
1 drug	62 (28.4)	22.5–34.4
2 drugs	55 (25.5)	19.5–31.0
≥3 drugs	74 (33.9)	27.7–40.2
**Duplicities**		
0 duplicity	196 (89.9)	85.9–93.9
≥1 duplicities	22 (10.1)	6.1–14.1
**Severe/moderate DDIs**		
0 severe/moderate DDIs	99 (45.4)	38.8–52.0
1 severe/moderate DDIs	45 (20.6)	15.3–26.0
≥2 severe/moderate DDIs	74 (33.9)	27.7–40.2

CCI: Charlson comorbidity index; CI: Confidence interval; DDIs: drug-drug interactions.

**Table 2:** Factors associated with PIM and PPO criteria among residents in the NH studied (reference category: non-PIM).

Demographic and Clinical Characteristics	Any v2 STOPP PIM	Any 2019 Beers PIM	Any PRISCUS PIM	Any v2 START PPO
	Odds Ratio (95% CI)			
**Sex**				
Male	3.65 (0.06–231.04)	0.32 (0.09–1.07)	0.65 (0.33–1.30)	1.41 (0.72–2.76)
**Age (years)**				
80–89	4.26 (0.05–405.61)	1.35 (0.35–5.25)	0.96 (0.38–2.39)	1.25 (0.53–2.94)
≥90	35.91 (0.14–9369.31)	2.34 (0.41–13.22)	0.77 (0.27–2.19)	1.33 (0.49–3.63)
**Pathologies**				
6–10	1.47 (0.02–114.36)	0.97 (0.24–3.98)	1.0 (0.42–2.36)	2.86 (1.34–6.07) *
≥11	6.89 (0.04–1237.31)	0.19 (0.03–1.28)	1.52 (0.54–4.25)	6.58 (2.32–18.68) *
**CCI**				
≥5	0.02 (0–1.45)	0.74 (0.16–3.41)	0.83 (0.32–2.19)	1.41 (0.59–3.37)
**Polypharmacy**				
Polypharmacy	11.22 (0.29–438.60)	14.06 (2.89–68.53) *	5.45 (1.60–18.63) *	1.76 (0.72–4.28)
Excessive polypharmacy	-	16.93 (1.08–265.76) *	8.42 (1.95–36.39) *	1.89 (0.55–6.50)
**Unnecessary drug**				
1 drug	-	1.24 (0.29–5.32)	0.40 (0.13–1.23)	1.17 (0.41–3.34)
2 drugs	-	3.15 (0.43–23.43)	0.53 (0.17–1.67)	0.92 (0.30–2.84)
≥3 drugs	-	2.02 (0.25–16.23)	0.64 (0.21–1.98)	1.17 (0.37–3.65)
**Duplicities**				
≥1 duplicities	-	0.22 (0.01–3.89)	0.72 (0.26–2.0)	0.98 (0.34–2.84)
**Severe/moderate DDIs**				
1 severe/moderate DDIs	1.86 (0.04–91.76)	6.62 (0.95–46.32)	0.75 (0.31–1.80)	1.23 (0.53–2.86)
≥2 severe/moderate DDIs	-	16.47 (1.09–249.77) *	2.01 (0.92–4.41)	1.06 (0.48–2.34)

* significant differences (*p* ≤ 0.05). CCI: Charlson comorbidity index; CI: Confidence interval; DDIs: drug-drug interactions; PIM: potentially inappropriate medication; PPO: potential prescribing omission.

**Table 3:** Multivariate ordinal logistic regression analysis of risk factors relevant to higher PIM according to STOPP criteria.

Variables	OR (95% CI)	*p*-Value
Age (70–79 years)	0.472 (0.231–0.964)	0.039
Pathologies	1.70 (1.102–2.623)	0.017
Polypharmacy	3.032 (1.698–5.416)	<0.001
Unnecessary drug	4.8 (3.368–6.854)	<0.001
Severe/moderate DDIs	1.837 (1.296–2.603)	0.001

CI: Confidence interval; DDIs: drug-drug interactions; OR: Odds Ratio; PIM: potentially inappropriate medication.

**Table 4:** Multivariate ordinal logistic regression analysis of risk factors relevant to higher PIM according to Beers criteria.

Variables	OR (95% CI)	*p*-Value
Sex (female)	2.587 (1.441–4.642)	0.001
Polypharmacy	3.158 (1.926–5.178)	<0.001
Severe/moderate DDIs	4.617 (3.172–6.718)	<0.001

CI: Confidence interval; DDIs: drug-drug interactions; OR: Odds Ratio; PIM: potentially inappropriate medication.

**Table 5:** Multivariate ordinal logistic regression analysis of risk factors relevant to higher PIM according to PRISCUS list.

Variables	OR (95% CI)	*p*-Value
Polypharmacy	2.579 (1.524–4.366)	<0.001
Severe/moderate DDIs	1.474 (1.039–2.091)	0.03

CI: Confidence interval; DDIs: drug-drug interactions; OR: Odds Ratio; PIM: potentially inappropriate medication.

**Table 6:** Prevalence, sensitivity, specificity, and level of consistency among the criteria applied in this study.

	v2 STOPP	2019 Beers	PRISCUS
PIM prevalence	96.3% (93.8–98.8)	90.8% (87.0–94.7)	35.3% (29.0–41.7)
Sensitivity (95% CI)	Reference	90.8% (88.8–96.0)	36.2% (29.7–42.7)
Specificity (95% CI)	Reference	50.0% (16.8–84.3)	87.5% (56.0–99.7)
Kappa Index (*p*-value)	Reference	0.25 (<0.001)	0.03 (0.169)

CI: Confidence interval; PIM: potentially inappropriate medication.

## Data Availability

Data are available on request from the corresponding author.
